# The Potential Role of Timosaponin-AIII in Cancer Prevention and Treatment

**DOI:** 10.3390/molecules28145500

**Published:** 2023-07-19

**Authors:** Zhaowen Liu, Yifan Cao, Xiaohua Guo, Zhixi Chen

**Affiliations:** College of Pharmacy, Gannan Medical University, Ganzhou 341000, China; liuzhaowenyifan@126.com (Z.L.); 13970722766@163.com (Y.C.); xhguo20041@163.com (X.G.)

**Keywords:** steroid saponin, Timosaponin-AIII, anticancer, apoptosis, autophagy, angiogenesis, multidrug resistance

## Abstract

Cancer, as one of the leading causes of death worldwide, has challenged current chemotherapy drugs. Considering that treatments are expensive, alongside the resistance of tumor cells to anticancer drugs, the development of alternative medicines is necessary. *Anemarrhena asphodeloides* Bunge, a recognized and well-known medicinal plant for more than two thousand years, has demonstrated its effectiveness against cancer. Timosaponin-AIII (TSAIII), as a bioactive steroid saponin isolated from *A. asphodeloides*, has shown multiple pharmacological activities and has been developed as an anticancer agent. However, the molecular mechanisms of TSAIII in protecting against cancer development are still unclear. In this review article, we provide a comprehensive discussion on the anticancer effects of TSAIII, including proliferation inhibition, cell cycle arrest, apoptosis induction, autophagy mediation, migration and invasion suppression, anti-angiogenesis, anti-inflammation, and antioxidant effects. The pharmacokinetic profiles of TSAII are also discussed. TSAIII exhibits efficacy against cancer development. However, hydrophobicity and low bioavailability may limit the application of TSAIII. Effective delivery systems, particularly those with tissue/cell-targeted properties, can also significantly improve the anticancer effects of TSAIII.

## 1. Introduction

Cancer, which is characterized by the dysregulated growth and proliferation of cells, has become one of the main causes of death worldwide. It is estimated that the number of new cancer cases increased by 19.3 million in 2020 and may continue to increase to 30.2 million by 2040 [1]. The World Health Organization (WHO) has reported that there will be 27 million new cancer cases and 17.1 million deaths by 2050 [2]. Extensive investigations and understanding of the pathogenesis of cancers have been advanced. Treatment options, such as surgery, chemotherapy, and radiotherapy, have been widely used in clinics. However, current strategies for cancer treatment are unsatisfactory due to their limited safety and effectiveness [1]. Developing efficient and safe chemotherapy agents for cancer treatment is still needed. The anticancer effects of chemotherapy agents could be associated with the disruption of the cell cycle, the induction of apoptosis, the regulation of autophagy, suppression of angiogenesis, the inhibition of migration and invasion, and the improvement of multidrug resistance [3,4].

**Figure 1:**
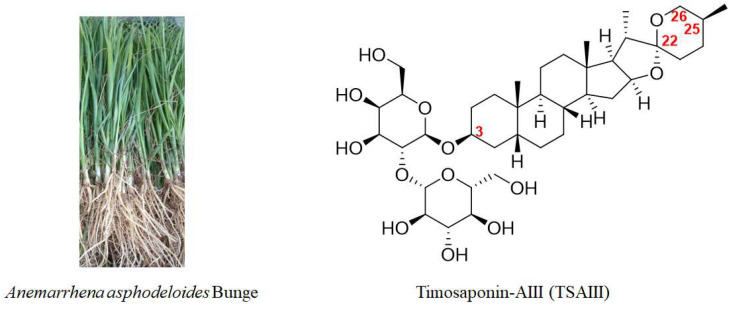
A picture of *Anemarrhena asphodeloides* Bunge and the chemical structure of TSAIII.

Natural products display a role in the prevention and treatment of cancers, and these can be further developed as novel candidates or anticancer agents. Bioactive compounds derived from natural products and primarily traditional medicinal plants are commonly used worldwide [5]. *Anemarrhena asphodeloides* Bunge (Figure 1), also known as Zhimu in Chinese, has been frequently used in traditional medicine in China, Japan, and Korea to treat various diseases, such as diabetes, hematochezia, arthralgia, coughs, and hemoptysis [6]. A phytochemical investigation into Zhimu has revealed that steroid saponins, phenylpropanoids, flavonoids, alkaloids, anthraquinones, and organic acids are its main constituents [6]. In addition, steroid saponin constituents, such as Timosaponin-AIII (TSAIII, Figure 1), Timosaponin-BII (TSBII), sarsasaponin, xanthones, and anemarsaponin, are one of the natural compound classes. Steroid saponins structurally consist of a lipophilic steroidal derivative and a hydrophilic glycoside moiety. Due to different sugar moiety substituents and sapogenins, saponins have diverse compounds [7].

Pharmacological assays for screening bioactive ingredients in Zhimu show that TSAIII is one of the natural steroidal saponins with multiple biological activities, such as antipyretics, anti-inflammation, antioxidation, antiplatelet aggregation, and antidepression [8]. Importantly, TSAIII has become one of the markers for the quality control of various *A. asphodeloides* Bunge-containing formulae, including Guizhi/Shaoyang/Zhimu formula, Zhimu/Bihe formula, TongGuan Wan, and *Rhizoma anemarrhenae*/*Phellodendron* formula [6,9,10,11,12]. Natural saponins have been demonstrated to have anticancer effects [13], and TSAIII has shown efficacy in different diseases, including Alzheimer’s disease (AD), diabetes, colitis, and cancer [8,14,15,16]. TSAIII has been proposed as a potent anticancer agent [8,9]. Recently, research on anticancer drugs has entered the fast lane. Informative data on the pharmacological activities of TSAIII are explosive. In this article, the anticancer effects of TSAIII are comprehensively updated.

## 2. Chemical Structure, Biotransformation, and Structure–Activity Relationship of TSAIII

TSAIII (Figure 1) has an IUPAC name: (2S,3R,4S,5S,6R)-2-[(2R,3R,4S,5R,6R)- 4,5-dihydroxy-6-(hydroxymethyl)-2-[(1R,2S,4S,5′S,6R,7S,8R,9S,12S,13S,16S,18R)-5′,7,9,13- tetramethylspiro [5-oxapentacyclo [10.8.0.02,9.04,8.013,18]icosane-6,2′-oxane]-16-yl] oxyoxan-3-yl]oxy-6-(hydroxymethyl)oxane-3,4,5-triol), which is a molecular formula of C_39_H_64_O_13_, and it has a molecular weight of 740.92 g/mol. TSAIII is a white to off-white solid with various pharmacological activities and is soluble in methanol, butanol, 80% ethanol, and aqueous pentanol; however, it is insoluble in water.

**Figure 2:**
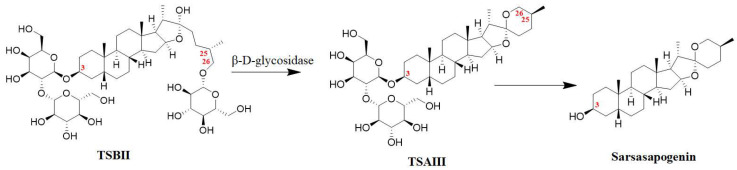
The biotransformation of TSAIII: the transformation of TSBII into TSAIII could be regulated by β-D-glycosidase, and TSAIII could be further transformed into sarsasapogenin by removing the sugar chain at the C-3 position.

It has been reported that the content of TSAIII in the rhizomes of *A. asphodeloides* is approximately 0.19–0.28% [17]. However, another study reported that the content of TSAIII is too low to be detected in a Zhimu/Baihe formula [18]. Interestingly, TSBII has a much higher level and can be transformed into TSAIII. β-D-glycosidase, as a key enzyme, has been used to produce TSAIII by hydrolyzing TSBII (Figure 2) from the crude extract liquid of the rhizomes of *A. asphodeloides*. Potentially, approximately 7 g of TSAIII can be isolated from 1 kg of the rhizomes of *A. asphodeloides* by five-step preparation. The optimal conditions for this include pH4.0, 55 °C, 2 h, and 600 U/g of β-D-glycosidase [17]. TSBII can be hydrolyzed and isomerized by the fungus *Colletotrichum gloeosporioides* to generate TSAIII, and TSAIII can also be produced by *Aspergillus niger* [19]. However, TSAIII could be further transformed into sarsasapogenin by removing the sugar chain at the C-3 position (Figure 2); this could induce a low yield of TSAIII [9,20]. Thus, an efficient industrial process for TSAIII production is essential.

The sugar chain at the C-3 position in TSAIII is indispensable to its pharmacological effects. The IC_50_ values of TSAIII and sarsasapogenin in lowing Aβ_42_ production in N2A-APPswe cells are 2.3 μM and 53 μM, respectively. In addition, disaccharide TSAIII has shown higher activity in reducing the production of Aβ_42_ compared with monosaccharide TSAI (IC_50_ = 6.1 μM). However, trisaccharide TSAV (IC_50_ = 4.2 μM) exhibits a lower activity than TSAIII [21]. Interestingly, an intact F ring is essential for TSAIII and sarsasapogenin to reduce Aβ_42_ production [21]. One study reported that an α-_D_-1,4-glucopyranosyl group at the C-4-OH of the glucosyl group in TSAIII could decrease the cytotoxicity of HL60 cells [22]. A structure–activity relationship study showed that the substituents at the C-3 and C-26 positions played a key role in selective anticancer activity [23,24]. Specifically, linking an amine (N-methyl piperazinyl, piperidyl, piperzinyl, pyrrolidinyl, N,N-dimethylamino, and N,N-diethylamino) to the C-3 or C-26 position and presenting a methoxyl group at the C-3 position could increase the selective cytotoxic activity of sarsasapogenin. In addition, the opening of the F ring in sarsasapogenin might enhance the antiproliferative activity [23].

## 3. Pharmacokinetic Profiles of TSAIII

Pharmacokinetic studies have exhibited a crucial role in novel drug development. The detection of TSAIII in animal blood plasma after the oral administration of TSAIII or TSAIII-containing formulae has been performed. After the oral administration of free TSAIII (6.8 mg/kg) in healthy male SD rats, the Cmax, Tmax, AUC0-t, and T1/2 of TSAIII were 18.2 ± 3.1 ng/mL, 2.3 ± 0.57 h, 150.5 ± 29.2 ng·h/mL, and 4.9 ± 2.0 h, respectively [25]. Another study reported that the values of T1/2, Cmax, and AUC were 2.74 ± 1.68 h, 105.7 ± 14.9 ng/mL, and 921.8 ± 289.0 ng·h/mL after the intragastrical administration of TSAIII (25 mg/kg) in male SD rats [26]. Comparably, the values of these were 15.1 ± 2.3 h, 77.28 ± 21.47 ng/mL, and 916.61 ± 208.43 ng·h/mL after the intragastrical administration of sarsasapogenin (25 mg/kg) [27]. Interestingly, these parameters for TSAIII could be 22.2 ± 6.5 ng/mL, 3.15 ± 0.62 h, 206.0 ± 45.1 ng·h/mL, and 9.9 ± 2.8 h, respectively, after the oral administration of Zhimu/Baihe formula (containing 6.4 mg/kg TSAIII). The improved pharmacokinetic profiles might be associated with the increased biotransformation of TSAIII and synergistic effects [25].

After the oral administration of the saponin extract from *Rhizoma anemarrhenae*, eight saponins were detected in the rat plasma. The pharmacokinetic profiles showed that TSAIII had the highest values of Tmax (7.85 h) and t1/2 (9.77 h). These suggested that TSAIII had a long body residence time and slow excretion [28]. Another study reported that after an oral administration of the 7 g/kg Zhimu/Bai formula, the level of TSAIII in the portal vein plasma and the systemic plasma in male Wistar rats was 1656.7 ± 121.1 ng/mL/h and 650.5 ± 45.2 ng/mL/h, respectively, indicating 60.7% of the liver extraction rate and the dramatic liver first-pass effect. The values of Cmax, Tmax, and t1/2 were 5085.3 ± 1581.5 ng/mL, 6 h, and 12.1 h, respectively. These suggested the comparable possibility of liver accumulation in TSAIII [18]. It has also been reported that Cmax, Tmax, and T1/2 of TSAIII were 94.4 ± 3.8 ng/mL, 336.0 ± 53.7 min, and 211.8 ± 98.8 min after the oral administration of the Zhimu/Bai formula (10 g/kg, containing 19.2 mg/kg of TSAIII) in male SD rats. In the other group, these values were 84.3 ± 8.6 ng/mL, 312 ± 65.7 min, and 104.2 ± 10.3 min after the oral administration of the Zhimu extract (10 g/kg, containing 18.7 mg/kg of TSAIII) [29].

The metabolites of TSAIII, including deglycosylated, glycosylated, and hydroxylated products, were identified, and these could be detected in the heart, urine, and feces. Only the parent form was detected in the plasma, liver, and kidney [30]. Another study reported that 19 metabolites were detected and identified from the rat plasma, bile, urine, and feces after a single oral dose of 400 mg/kg of TSAIII. Consistently, TSAIII underwent deglycosylation, oxidation, dehydrogenation, F-ring cleavage, and isotype reactions [31]. Salt processing, as a traditional processing method for Chinese herbs, has been reported to increase the content of TSAIII due to its increased transformation from TSBII. The AUC and Cmax of TSAIII after the oral administration of extracts of the unprocessed *A. rhizoma* were 16.5 μg·h/L and 4.5 μg/L, respectively. However, these values decreased to 7.2 μg·h/L and 2.18 μg/L, respectively, after the oral administration of the processed samples. The explanation for these discrepancies in the pharmacokinetic parameters of TSAIII is complex. For example, salt processing could increase the metabolism and excretion of TSAIII [32].

It has been reported that hydrophobicity and low bioavailability limit the antitumor efficacy and application of TSAIII. Indeed, new delivery systems for TSAIII have been developed. The anti-CD44 antibody, as a tumor-target ligand, has been used to improve liposome accumulation in tumors by specifically interacting with CD44 receptors. It has been reported that anti-CD44 antibody-modified TSAIII-loaded liposomes (CD44-TSAIII-LP) could increase the circulation time, the bioavailability, the tumor-target accumulation, and the antitumor activity of TSAIII, demonstrating a potential agent for the therapeutic management of CD44-positive cancers [33]. A grapheme oxide-based nanocomposite hydrogel (GPP) has been developed to improve the bioavailability of TSAIII, enhance the efficacy of photothermal therapy, and elevate its protective activity against tumor development. Specifically, the encapsulation rate of GPP-TSAIII is 66.36%, and this delivery system was found to have a slower release and higher uptake of TSAIII in mouse melanoma B16F10 cells in vitro [34].

## 4. The Anticancer Effects of TSAIII

**Figure 3:**
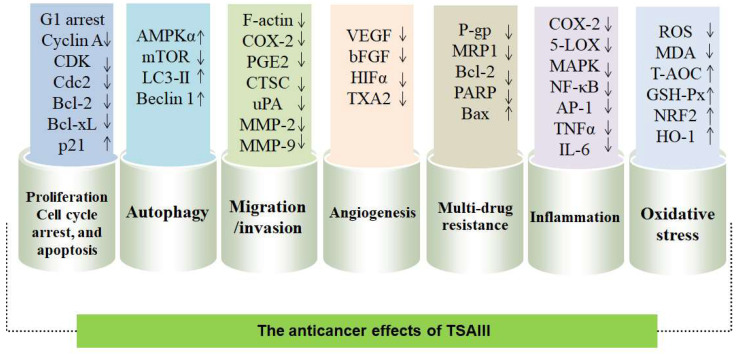
The anticancer effects of TSAIII are shown: TSAIII inhibits proliferation, causes cell cycle arrest, induces apoptosis, mediates autophagy, inhibits migration and invasion, attenuates angiogenesis, suppresses multidrug resistance, decreases inflammation, and ameliorates oxidative stress in cancer cells. ↑ indicates up-regulation, and ↓ indicates down-regulation.

The anticancer effects of TSAIII have been associated with its capability to inhibit proliferation, cause cell cycle arrest, induce apoptosis, mediate autophagy, suppress migration and invasion, attenuate angiogenesis, and reverse multidrug resistance (Figure 3). In addition, TSAIII has exhibited inhibitory activity against inflammation, oxidative stress, and carcinogenesis. TSAIII shows the cytotoxicity of various cell types in different models by affecting numerous cellular signaling pathways both in vivo and in vitro. TSAIII specifically inhibits growth and viability in cancer cells but not normal cells [20]. Thus, TSAIII has been proposed as a potent anticancer agent.

### 4.1. Proliferation Inhibition, Cell Cycle Arrest, and Apoptosis Induction

One of the most dominant characteristics of tumor cells is uncontrolled proliferation, which could be due to the dysregulation of the cell cycle and its resistance to apoptotic cell death. The cell cycle at each cellular phase was tightly mediated by cyclin-dependent kinases (CDKs). Specifically, CDK/cyclin complexes and CDK inhibitors govern the progression of the cell cycle at each phase. The induction of cell cycle arrest could result in the inhibition of cell proliferation [35]. In addition, the induction of apoptosis or programmed cell death is also an effective way to control the proliferation of cancer cells [36]. TSAIII has shown regulatory effects against tumor pathogenesis. For example, TSAIII can inhibit proliferation, cause cell cycle arrest at the G1 phase, and promote caspase-dependent apoptosis by mediating the PI3K/AKT signaling pathway in pancreatic cancer cells [37]. TSAIII also induces caspase-dependent apoptosis in promyelocytic leukemia HL60 cells by enhancing the phosphorylation levels of JNK1/2 and p38 MAPK [38]. Another study reported that TSAIII downregulates Cyclin B1 Cdc2, and Cdc25C expression, triggers DNA damage, causes cell cycle arrest at the G2/M phase, and induces apoptosis in breast cancer MDA-MB-231 and MCF7 cells by activating the ATM/Chk2 and p38 MAPK signaling pathways [39] (Table 1).

The signal transducer and activator of transcription 3 (STAT3) are constantly activated via Ser705 phosphorylation to promote G1/S-phase progression and enhance the proliferation of pancreatic cancer cells. Potentially, TSAIII can suppress the phosphorylation of STAT3 and ERK1/2, decrease cell viability, cause cell cycle arrest at the G1 phase, and promote cell apoptosis in AsPC-1 cells by inhibiting the constitutive expression of c-Src kinase, which is frequently and aberrantly activated in various malignancies. Specifically, TSAIII has been shown to significantly decrease the expression of Bcl-2, Bcl-xL, MMP-9, VEGF-1, and survivin and increase the expression of the cyclin-dependent kinase inhibitor p21 and cell cycle regulator cyclin D1 [40]. In human colorectal cancer HCT-15 cells, TSAIII has shown an inhibitory effect with an IC50 value of 6.1 μM. TSAIII suppresses proliferation, causes cell cycle arrest at the G0/G1 and G2/M phase, and induces apoptosis, as demonstrated by the downregulated expression of cyclin A, cyclin B1, CDK2, CDK4, the proliferating cell nuclear antigen, c-Myc, Bcl-2, and Bcl-xL and increased DNA fragmentation, caspase activation, and cleaved poly-(ADP ribose) polymerase in HCT-15 cells. Consistently, TSAIII has been shown to inhibit tumor growth in HCT-15-xenograft-bearing mice [41] (Table 1).

Further studies have shown that TSAIII induces mitochondria-mediated and caspase-dependent apoptosis in HepG2 cells, as shown by the increased expression of caspase-3, caspase-7, caspase-7, and caspase-9, the decreased expression of Bcl-2, Mcl-1, cIAP-1, cIAP-2, XIAP, survivin, and livin, and the enhanced cytosolic production of HtrA2/Omi, Smac/Diablo, and cytochrome c. In addition, TSAIII reduces the viability of HepG2 cells with an IC50 value of 15.41 μM [42]. The Ras-associated protein 1 (RAP1) is a small GTPase that is responsible for inside-out signaling cascades to trigger integrin [43]. The aberrant expression of RAP1 can be associated with aggressive phenotypes and tumor invasion and metastasis, while the GTPase-activating protein RAP1GAP can negatively regulate the activity of RAP1 [44]. It has been reported that TSAIII strengthens the suppressive activity of paclitaxel (PTX) on the proliferation of nasopharyngeal carcinoma CNE-1 and HNE-2 cells, upregulates the expression of Bad and RAP1GAP, downregulates the expression of Bcl-2, RAP1, and RasGRP2, and promotes PTX-induced apoptosis. TSAIII can synergize with PTX to protect against tumor growth in a xenograft mouse model [45] (Table 1).

The cGMP/PKG signaling pathway is downregulated in the glioblastoma multiforme (GBM), and the activation of the cGMP/PKG pathway has been shown to inhibit the progression of GBM [46]. TSAIII promotes cell apoptosis and inhibits the growth of human glioblastoma U87MG cells, which is the deadliest malignant tumor in the brain. Mechanically, TSAIII downregulates the expression of cGMP-specific phosphodiesterase 5 (PDE5), upregulates the expression of guanylate cyclases (sGCβ), cGMP, PKG, and VASP-Ser239 phosphorylation, and suppresses the Wnt/β-catenin signaling pathway [47]. Sterol regulatory element-binding proteins (SREBPs) mediate the synthesis of fatty acids and cholesterol. In the endoplasmic reticulum (ER), SREBP can be inactivated by forming a complex with INSIG-1 and the SREBP cleavage-activating protein (SCAP). Active SREBP-1 mediates fatty acid synthesis, and SREBP-2 regulates cholesterol synthesis by activating target genes, such as HMG-CoA reductase (HMGCR) [48,49]. It has been reported that TSAIII inhibits the expression of SREBP-1, FASN, ACC, and HMGCR and suppresses the growth of pancreatic cancer BxPC-3 cells. In addition, TSAIII causes cell cycle arrest at the G0/G1 phase, decreases the expression of cyclin E1, cyclin D1, CDK2, and CDK6, increases the expression of p21 and p27, and upregulates the expression of cleaved caspase-3, cleaved caspase-9, cleaved PARP, and Bid [50] (Table 1).

Cholesterol-based liposomes in the formulation have been approved for drug development. However, cholesterol is vulnerable to oxidation, which negatively affects the stability and quality of liposomes. TSAIII has a similar structure to cholesterol. It has been reported that TSAIII-based TSAIII/DOX codelivery liposomes have been developed, and TSAIII acts as a bilayer stabilizer, increasing the doxorubicin hydrochloride (DOX) uptake and synergizing with DOX to induce apoptosis in HepG2 and HCC-LM3 cells [51]. CCR4-NOT2 (CNOT2) plays a key role in apoptosis, autophagy, proliferation, and angiogenesis in various tumor cells [52]. Midline1 interacting protein 1 (MID1IP1), an upstream factor of AMPK, mediates acetyl-CoA carboxylase activity and acts as an oncogene [53]. It has been reported that deficiency of both MID1IP1 and CNOT2 can promote cell growth and inhibit the apoptosis of hepatocellular carcinoma HepG2 and Huh7 cells by downregulating the expression of c-Myc [54]. TSAIII can induce colon cancer HCT116 cell apoptosis by suppressing the expression of MID1IP1, CNOT2, and c-Myc, interrupting the interaction between MID1IP1 and c-Myc and decreasing the stability of c-Myc. The cotreatment of TSAIII with 5-FU or doxorubicin can attenuate c-Myc and caspase-3 expression and increase cleaved-PARP production, potentiating the apoptotic effects of 5-FU or doxorubicin in HCT116 cells [55] (Table 1).

### 4.2. Autophagy Mediation

Autophagy can be associated with the selective degradation of aggregated protein complexes and damaged organelles, which serve as a cellular quality control system. Attenuated or impaired autophagy has been related to carcinogenesis, and the activation of autophagy has become a therapeutic strategy for cancers [56]. TSAIII can be an inducer of autophagy and stimulate autophagosome formation and autophagy flux by inhibiting the mTOR/ULK1 signaling pathway and elevating cytosolic-free calcium. TSAIII promotes autophagy-mediated protein degradation, compromising the rapid growth of tumor cells [57].

**Figure 4:**
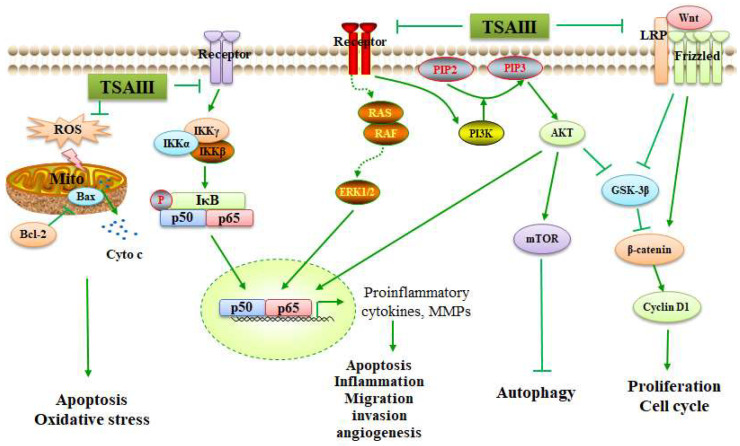
TSAIII inhibits cancer development by mediating various signaling pathways: TSAIII can inhibit the NF-κB, ERK1/2, PI3K/AKT, and Wnt/β-catenin signaling pathways. TSAIII induces mitochondria-dependent apoptosis and suppresses oxidative stress. The NF-κB pathway-mediated upregulation of proinflammatory cytokines and MMPs has been associated with cancer cell apoptosis, inflammation, migration, invasion, and angiogenesis. In addition, the activation of the PI3K/AKT/mTOR pathway inhibits autophagy. The inactivation of the GSK-3β and activated β-catenin/Cyclin D1 pathway could lead to cell proliferation and cell cycle mediation.

The interaction between autophagy and apoptosis is complex during tumorigenesis progression. Members in the inhibitor of the apoptosis (IAP) family can bind to caspases and inhibit their activity in eukaryotic cells. XIAP, as an identified inhibitor of the apoptosis (IAP) protein, is a potential target for HCC treatment. TSAIII reduces cell viability and exhibits cytotoxicity to HCC cell lines in a p53-independent manner. The inhibition of XIAP promotes caspase-dependent apoptosis in TSAIII-treated HCC cells. Interestingly, the mRNA expression of XIAP is not changed, and TSAIII promotes autophagy-induced XIAP degradation by activating the AMPKα/mTOR signaling pathway. The ubiquitination of XIAP is required for TSAIII-induced XIAP protein degradation. However, the recruitment of the E3 ligase RING has not been observed, indicating an impaired proteasomal degradation system. Therefore, the inhibition of autophagy switches on necrosis in TSAIII-treated HCC cells [58]. Another study has reported that TSAIII upregulates LC3-II expression and induces autophagy at an early stage in HeLa cancer cells. Prolonged exposure to TSAMII can lead to the overproduction of reactive oxygen species (ROS), a reduction in mitochondrial membrane potential (ΔΨm), the release of cytochrome c, and activation of caspase-3 (Table 1) (Figure 4). The inhibition of autophagy by 3-methyladenine (3-MA) or Beclin 1 siRNA enhances the cytotoxic sensitivity of HeLa cells to TSAIII in a caspase-dependent manner. Thus, TSAIII-induced autophagy precedes mitochondria-dependent cell apoptosis [59].

Consistently, TSAIII induces mitochondrial dysfunction, upregulates the protein expression of cleaved caspase-3, cleaved caspase-9, cleaved PARP, and cytochrome c, downregulates the expression of Mcl-1, and enhances the accumulation of p62, LC3-II, and LAMP1 in GBM8401 and M059K cells. The inhibition of autophagy by 3-MA enhances TSAIII-induced apoptosis. In addition, TSAIII has been shown to suppress tumor growth in GBM8401-xenograft-bearing mice and orthotropic glioma mice [60]. In human non-small-cell lung cancer (NSCLC) A549 and H1299 cells, TSAIII at higher concentrations (10 μM and 30 μM) has promoted both autophagy and apoptosis by activating AMPK signaling and inactivating the ERK1/2 pathway (Table 1). At a lower concentration (1 μM), TSAIII only induces autophagy, protecting cancer cells from apoptosis. In addition, a combination of TSAIII with 3-MA promotes cell apoptosis and enhances their anticancer effects [61].

It has also been reported that TSAIII promotes both apoptosis and autophagy in A375-S2 cells. The activation of autophagy suppresses apoptosis in TSAIII-treated A375-S2 cells. In addition, TSAIII can initiate cell apoptosis by enhancing the phosphorylation levels of JNK and ERK [62]. Similarly, TSAIII upregulates the Bax expression, downregulates Bcl-2 expression, and promotes Beclin 1 and LC3-II accumulation by suppressing the PI3K/AKT/mTOR signaling pathway in T-cell acute lymphoblastic leukemia Jurkat cells [63] (Figure 4). TSAIII can activate caspase-4 expression, induce cell apoptosis, and promote autophagy by inhibiting mTORC1 expression and PERK/eIF2α signaling in MDA-MB-231 and BT474 cells. TSAIII stimulates protective autophagy and proapoptotic pathways, while the inhibition of autophagy has been shown to contribute to TSAIII-induced apoptosis [20].

### 4.3. Suppression of Migration and Invasion

Metastasis is a complex process that includes the invasion/metastasis cascade [64]. Integrins, constituted by α and β subunits, interact with the extracellular matrix (ECM) and trigger the recruitment of actin filaments and their related factors in signaling pathways to integrins. Vital factors, such as focal adhesion kinase (FAK), AKT, Rho GTPase, and the Src family kinase, are activated [65]. The reorganization of actin filaments mediates downstream factors, such as cofilin, LIM domain kinase (LIMK), and testis-associated actin remodeling kinase 1 (TESK1) [66]. It has been reported that TSAIII could significantly reduce the expression and distribution of F-actin, which plays a key role in cancer cell migration and invasion. Mechanically, TSAIII suppresses the invasion of human osteosarcoma cells by downregulating the expression of integrin-αv, integrin-β3, and the phosphorylation of FAK/Src and upregulating the expression of TESK1 and the phosphorylation of cofilin, which results in the destruction of the F-actin cytoskeleton [67].

Cyclooxygenase (COX) has two isoforms, COX-1 and COX-2. COX-1 plays a key role in maintaining cellular homeostasis; COX-2 is an inducible enzyme that can be associated with inflammation, invasion, and migration [68]. It has been reported that the overexpression of COX-2 and its metabolite PGE2 promotes tumor progression and metastasis [69]. TSAIII treatment may result in the inhibition of cell migration by inhibiting the NF-κB signaling pathway and downregulating the expression of COX-2, PGE2, and PGE2 receptors (EP2 and EP4) in B16-F10 cells [70] (Table 1) (Figure 4). CTSC, as a cysteine protease in the cathepsin family, can be highly correlated with tumor metastasis. TSAIII has been reported to inhibit cell viability, migration, and invasion by suppressing the/AKT signaling and downregulating the CTSC expression in renal carcinoma 786-O and A-498 cells. CTSC can be a direct target of miR-129-5p, and both the specific PI3K inhibitor LY294002 and miR-129-5p can downregulate the expression of CTSC and inhibit the metastasis of 786-O and A-498 cells [71] (Table 1).

Cervical cancer is the most common gynecologic tumor worldwide. The remodeling of ECM facilitates the migration and invasion of cancer cells, and the urokinase-type plasminogen activator (uPA) is the critical factor that is responsible for mediating ECM degradation [72]. TSAIII can inhibit migration and invasion by suppressing p38 MAPK signaling and uPA expression. The knockdown of p38 synergistically facilitates TSAIII-inhibited uPA expression and metastasis in human cervical cancer SiHa and HeLa cells. In addition, TSAIII significantly reduces the lung metastasis nodule number, lung weight, and tumor mass and improves pathological changes [73]. There are more than 20 MMPs that are responsible for the degradation of ECM. Among these, MMP-2 and MMP-9 are the two major enzymes for degrading gelatin. It has been reported that TSAIII downregulates the expression of MMP-2 and MMP-9 and inhibits the migration and invasion of human non-small-cell lung cancer A549 cells by attenuating the ERK1/2, Src/FAK, and β-catenin signaling pathways [74] (Table 1).

B lymphoma Mo-MLV Insertion region 1 (BMI1), a polycomb group protein, is associated with the development of various malignancies, such as breast, leukemia, and prostate cancers [75]. The overexpression of BMI1 can result in bypassed cellular senescence and the increased replicative lifespan of cancer cells [76]. It has been reported that TSAIII can be an inhibitor of BMI1, inhibiting oncogenic phenotypes, such as proliferation, migration, and invasion, and inducing cellular senescence in MDA-MB-231 and MCF7 cells. In addition, TSAIII upregulates the expression of miR-200c and miR-141, which are tumor suppressors that are negatively mediated by BMI1 [77]. The hepatocyte growth factor (HGF) may upregulate the protein expression of COX-2 and MMP-9, increase the production of intracellular ROS, induce the activation of c-MET and ERK, and subsequently enhance the invasive activity of MDA-MB-231 cells (Table 1). However, TSAIII can abolish HGF-induced invasive effects [78].

A combination of ginsenoside Rg1 with TASIII exhibits synergistic cytotoxic effects against human osteosarcoma MG63 cells and induces caspase-dependent apoptosis. In addition, TSAIII, combined with Rg1, has shown antimetastatic activity, as indicated by the decreased expression of MMP-2 and MMP-9 in both MG63 and U2OS cells. This underlying mechanism might be associated with the decreased phosphorylation of JNK, ERK, and p38 MAPK and the attenuated interaction between β-catenin and CREB with the combination of Rg1 with TSAIII in MG63 cells [79]. Consistently, ginsenoside Rb1 and Rc also synergize with TSAIII to attenuate migration and invasion and increase the caspase-dependent apoptosis of MG63 cells by suppressing the MAPK, β-catenin/CREB, and FAK/Src signaling pathways [80].

### 4.4. Anti-Angiogenesis

Angiogenesis plays a key role in tumor growth and metastasis. The formation of new blood vessels can facilitate the transportation of oxygen and nutrients to the tumor cells, promoting the growth and metastasis of tumors. The angiogenic factors produced by tumor cells include the vascular endothelial growth factor (VEGF), basic fibroblast growth factor (bFGF), TNFα, and HIFα [81]. It has been reported that TSAIII suppresses cell viability and exhibits cytotoxicity in human umbilical vein endothelial cells (HUVECs). In addition, TSAIII decreases VEGF-induced proliferation, migration, invasion, and tube formation in HUVECs by downregulating the VEGF/PI3K/AKT/MAPK signaling pathway. Furthermore, TSAIII inhibits angiogenesis in the transgenic zebrafish line Tg (fli-1a: EGFP)y1, as shown by the decreased growth of intersegmental vessels (ISVs) and subintestinal vessels (SIVs) [82].

COX-2 can bind to thromboxane A2 (TXA2), which is highly overexpressed in vascularized solid tumors and promotes angiogenesis and metastasis by activating the PI3K/AKT signaling pathway [83]. Platelets comprise various angiogenesis-stimulating factors, and platelet activation can be associated with tumor metastasis [84]. It has been reported that TSAIII is a selective inhibitor of the TXA2 receptor and suppresses the overproduction of TXA2. The inhibition of TXA2 by TSAIII could lead to the attenuation of platelet aggregation and activation [85].

### 4.5. Reverse of Multidrug Resistance

Chemotherapy, as an effective strategy for metastatic tumor treatment, can often fail in the clinic due to the multidrug resistance (MDR) of cancer cells. The ATP-dependent transporter (ABC), encoded by the MDR1 or ABCB1 gene, is a phosphoglycoprotein named glycoprotein P (P-gp), which is the most crucial efflux transporter among the ABC transporter family. P-gp has a broad substrate spectrum, which impacts the clinical efficacy of various chemotherapeutic drugs [86,87]. It has been reported that TSAIII and sarsasapogenin can be substrates of P-gp. The efflux ratio of TSAIII is 2.26, which is higher than the accepted threshold of two for P-gp substrates. By contrast, the efflux ratio of sarsasapogenin is 1.51. Interestingly, the Papp (AP-BL) and Papp (BL-AP) of TSAIII enhance this, and the efflux ratio of TSAIII decreases from 2.26 to 0.63. The Papp (AP-BL) of sarsasapogenin increases while the Papp (BL-AP) decreases, and the efflux ratio of sarsasapogenin decreases from 1.51 to 0.87. This indicates that TSAIII can be either a substrate or an inhibitor of P-gp. Furthermore, TSAIII can decrease the efflux ratio of Rh-123 from 2.46 to 1.22 [88].

Another study has reported that TSAIII can reverse the multidrug resistance of human chronic myelogenous leukemia K562/ADM cells, as shown by the increased intracellular accumulation of ADM and decreased mRNA expression of P-gp and MRP1. Mechanically, the effects of TSAM on their reversal sensitivity to ADM might be associated with the inhibition of the PI3K/AKT signaling pathway [89] (Table 1). The overexpression of P-gp in A549/Taxol and A2780/Taxol cells has also been shown [90]. TSAIII can exhibit cytotoxicity and decrease the cell viability of A549/Taxol and A2780/Taxol cells with IC50 values of 5.12 μM and 4.64 μM, respectively. In addition, TSAIII upregulates the Bax protein expression, downregulates Bcl-2 and PARP expression, and promotes apoptosis in A549/Taxol and A2780/Taxol cells. Mechanically, the anticancer effects of TSAIII on taxol-resistant cells could be associated with the inhibition of PI3K/AKT/mTOR and Ras/Raf/MEK/ERK signaling pathways, which have become therapeutic targets for multidrug resistance [90] (Table 1).

### 4.6. Anti-Inflammation

Inflammation has been implicated in various diseases, such as cancer, obesity, diabetes, AD, and osteoarthritis. The activation of the arachidonic acid (AA) cascade results in the overexpression of COX-2 and 5-lipoxygenase (5-LOX). COX-2 and 5-LOX are two independent pathways that contribute to inflammatory responses. The inhibition of one of these pathways could switch the metabolism of AA to the other. The dual inhibition of the COX-2 and 5-LOX pathways might be effective for inflammatory diseases. TSAIII has been identified as the dual inhibitor of COX-2 and 5-LOX enzymes, with IC50 values of 1.81 μM and 1.21 μM, respectively [91]. Chronic inflammatory reactions participate in the pathogenesis of benign prostatic hyperplasia (BPH). Specifically, AA metabolism-associated COX-2 stimulates the occurrence and development of BPH by inducing local vasodilation and increasing vascular permeability. TSAIII is one of the bioactive components in the Zi-shen pill, which improves pathological changes in BPH by suppressing the COX-2/PGE2 signaling [92].

Consistently, another study has also shown that TSAIII is an effective compound in the Zi-shen pill, which has been demonstrated to inhibit 5-LOX activity. TSAIII can be a ligand of 5-LOX with a specific binding value of 2.919 ± 0.296 using affinity ultrafiltration UPLC-MS [93]. The NF-κB signaling pathway, consisting of p65 and p50, has been implicated in most inflammatory responses and natural immunity. Lipopolysaccharide (LPS) can promote the expression and secretion of proinflammatory cytokines, such as IL-1β and TNFα, by activating the TLR/NF-κB signaling pathway, and often results in inflammation [94]. TSAIII is one of the most effective compounds in the *A. asphodeloides* extract: it inhibits inflammatory responses and promotes immune effects by suppressing the MAPK and NF-κB signaling pathways in LPS-treated RAW 264.7 cells [95]. In scopolamine-treated neuroblastoma SK-N-SH cells, TSAIII has also shown inhibitory activity against the production of TNFα and the expression of NF-κB [96].

It has been reported that TSAIII and the gut microbiota-mediated metabolite sarsasapogenin exhibit anti-inflammatory effects by inhibiting the activation of NF-κB and MAPK alongside the phosphorylation of IRAK1, TAK1, and IκBα in LPS-treated macrophages. Specifically, TSAIII and sarsasapogenin inhibit the interaction of LPS with its receptor TLR4, with the polarization of M2 to M1 macrophages, the expression of IL-1β, TNFα, IL-6, COX-2, and PGE2, which results in the suppression of inflammatory responses [16]. In addition, sarsasapogenin also improves inflammatory responses and insulin resistance in adipose tissues of high fat diet fed obese mice by inhibiting macrophage infiltration. The underlying mechanism of sarsasapogenin in protecting against insulin-resistance-related diseases might be associated with the suppression of IKK/NF-κB and JNK signaling pathways in adipocytes [97]. The ethanol extract of *A. asphodeloides*, and particularly the saponin-enriched fraction, has demonstrated protective activity against LPS-induced acute lung injury, as shown by a decreased inflammatory cell number in the bronchoalveolar lavage fluid (BALF) and improved histological changes in the lung tissues. TSAIII reduces inflammatory cells in the BALF and decreases the production of proinflammatory cytokines, such as IL-6. Mechanically, TSAIII suppresses the activation of STAT3 but has not been shown to affect the MAPK, NF-κB, and AP-1 signaling pathways [98].

### 4.7. Antioxidant Effects

Chronic UVB radiation also results in sustained immunosuppression and photocarcinogenesis. It has been reported that TSAIII protects against the UVB-induced invasive activity of human epidermal keratinocytes (HEKs) and human dermal fibroblasts (HDFs) by downregulating the expression of MMP-2 and MMP-9 and upregulating the expression of TIMP-1 and TIMP-2. Mechanically, TSAIII suppresses UVB-induced MAPK, NF-κB, and AP-1 signaling pathways, leading to the inhibition of inflammatory responses. In addition, UVB radiation could result in oxidative injury in the DNA and promote the formation of the adduct 8-oxo-dG. TSAIII protects against UVB-induced DNA injury by downregulating the expression of DNA repair enzymes, such as PCNA and SMC1 [99].

Oxidative stress contributes to tumorigenesis. UVB-induced cell damage can be associated with the overproduction of ROS and its related signaling pathways, such as MAPK. It has also been reported that TSAIII protects against the UVB-induced generation of proinflammatory cytokines, the upregulation of MMP-1, and the downregulation of TIMP [100]. In high-fat-induced obese rats, TSAIII has been shown to decrease the levels of ROS and MDA, increase the production of T-AOC and GSH-Px, and improve oxidative stress by downregulating the protein expression of p-NF-κB and upregulating the protein expression of NRF2/heme oxygenase-1 (HO-1) [101]. Controversially, it has been reported that TSAIII (1–10 μM) treatment might dose-dependently increase the production of ROS, upregulate the expression of HO-1, and decrease the generation of ATP and the levels of mitochondrial membrane potential, indicating the induction of hepatotoxicity in rats. NAC (5 mmol/L) or mangiferin (10–20 μg/mL) can suppress TSAIII-induced ROS generation [102].

### 4.8. Miscellaneous

Ferroptosis is an iron-dependent form of cell death. The induction of ferroptosis can be a novel strategy for cancer treatment. It has been reported that TSAIII could induce iron accumulation, suppress cell proliferation, cause cell cycle arrest at the G2/M phase, inhibit migration, and trigger cell death in NSCLC H1299 and A549 cells, accompanied by the enhanced production of ROS and MDA. Mechanically, TSAIII can interact with HSP90 and form a complex to promote the ubiquitination and degradation of glutathione peroxidase 4 (GPX4) [103] (Table 1). TASIII is one of the bioactive components in the formula of Baihe Zhimu Tang, which could decrease the efficacy of tamoxifen against breast cancer and the concentrations of endoxifen and 4-OH-tamoxifen in tumor-bearing mice. This might be associated with the inhibitory activity of the formula against rat liver microsome-derived cytochrome P450 (CYP450), which induces the demethylation and oxidation of tamoxifen [104]. TSAIII and sarsasapogenin can inhibit immunoglobulin E (IgE)-induced passive cutaneous anaphylaxis (PCA) in mice. In addition, TSAIII is less potent in suppressing degranulation and IL-4 expression than sarsasapogenin in IgE-treated RBL-2H3 cells [105].

**Table 1:** The anticancer effects of TSAIII in different cancer types.

Cancer Types	Cell Lines	Concentrations	Key Molecular Targets or Signaling Pathways	Effects	Refs.
Pancreatic cancer	PANC-1, BxPC-3	5, 10, 20 μM	Cell cycle G1 arrest, p-BAD↓, p-mTOR↓, p-p70S6↓, cleaved caspase↑	Causes cell cycles, inhibits proliferation, and induces apoptosis	[37]
	AsPC-1	5, 20 μM	Cell cycle G1 and G2/M arrest, p-ERK1/2↓, p-STAT3↓, Apoptotic rate↑, p-c-Src kinase↓, Bcl-2↓, MMP-9↓, VEGF-1↓, cyclin D1↑, p21↑	Causes cell cycles, induces apoptosis, and inhibits proliferation, metastasis, and angiogenesis	[40]
	BxPC-3	2.5, 5 μM	Mature SREBP-1↓, FASN↓, ACC↓, HMGCR↓, cell cycle G0/G1 arrest, cyclin E1↓, cyclin D1↓, CDK2↓, CDK6↓, p21↑, p27↑, cleaved caspase-3↑, cleaved caspase-9↑, cleaved PARP↑, Bid↑	Causes cell cycles, induces apoptosis, and inhibits proliferation	[50]
Leukemia	HL60	2, 4, 8 μM	Apoptotic rate↑, cleaved caspase-3↑, caspase-8↑, caspase-9↑, and PARP↑, p-JNK1/2↑, p-p38↑	Induces apoptosis	[38]
	Jurkat	2, 8 μM	Proliferation↓, apoptotic rate↑, Bcl-2↓, Bax↑, LC3-II↑, Beclin 1↑, p-PI3K↓, p-AKT↓, p-mTOR↓	Inhibits proliferation and induces apoptosis and autophagy	[63]
	K562/ADM	1, 2 μM	Intracellular accumulation of ADM↑, P-gp↓, MRP1↓, p-AKT↓	Reverses drug resistance	[89]
Breast cancer	MDA-MB-231, BT474	5 μM	caspase-4↑, Bim↑, REDD1/DDIT4↑, p21CIP↑, stratifin↑, GDF15↑, Myc↓, Id1↓, Id3↓, mTOR↓, eIF2α↑, CHOP↑, LC3-II↑	Induces apoptosis and autophagy	[20]
	MDA-MB-231, MCF7	10, 15 μM	Cell cycle G2/M arrest, Cdc25C↓, CyclinB1↓, Cdc2↓, Ki67↓, PCNA↓, Bcl-2/Bax ratio↓, caspase-3↑, ATM↑, γ-H2AX↑, p-p38/p38↑	Causes cell cycles, inhibits proliferation, and induces apoptosis	[39]
	MDA-MB-231, MCF7	2, 4 μM	Proliferation↓, migration↓, invasion↓, BMI1↓, H2AUb↓, c-Myc↓, miR-200c↑, miR-141↑	Inhibits proliferation and inhibits migration and invasion	[77]
	MDA-MB-231	10^−8^, 10^−7^, 10^−6^ M	Invasion↓, COX-2↓, p-ERK↓, p-cMet↓, MMP-9↓, ROS↑	Inhibits invasion	[78]
Colon cancer	HCT-15	5, 10, 20 μM	IC_50_ = 6.1 μM, cell cycle G0/G1 and G2/M arrest, cyclin A↓, cyclin B1↓, CDK2↓, CDK4↓, proliferating cell nuclear antigen↓, c-Myc↓, Bcl-2↓, Bcl-xL↓, cleaved caspase-3↑, caspase-8↑, caspase-9↑, PARP↑	Causes cell cycles, inhibits proliferation, and induces apoptosis	[41]
	HCT116	7.5, 10, 12.5 μM	MID1IP1↓, CNOT2↓, c-Myc↓, c-Myc stability↓, caspase-3↑, PARP↑	Inhibits proliferation and induces apoptosis	[55]
Liver cancer	HepG2	6, 9, 12, 15 μM	IC_50_ = 15.41 μM, apoptotic rate↑, caspase-3↑, caspase-7↑, caspase-8↑, caspase-9↑, Bcl-2↓, Mcl-1↓, cIAP-1↓, cIAP-2↓, XIAP↓, survivin↓, livin↓	Induces mitochondria-mediated and caspase-dependent apoptosis	[42]
	HepG2 MHCC97L PLC/PRF/5 Hep3B	5, 10, 20 μM	Cleaved caspase-3↑, PARP↑, XIAP↓, AMPKα↑, mTOR↓, LC3-II↑, p-S6K↑, p-S6↑	Induces apoptosis and autophagy	[58]
Nasopharyngeal cancer	CNE-1, HNE-2	15 μM TSAIII + 8 Μm PTX	Apoptotic rate↑, Bad↑, RAP1GAP↑, Bcl-2↓, RAP1↓, RasGRP2↓	Inhibits proliferation and induces apoptosis	[45]
Glioma	U87MG	5, 10 μM	β-catenin↓, cyclin D1↓, Bcl-2↓, PDE5↓, sGCβ↑, cGMP↑, PKG↑, p-VASP-Ser239↑	Inhibits proliferation and induces apoptosis	[47]
	GBM8401 and M059K	5, 10, 15 μM	IC_50_ = 9.5 and 8.9 μM, respectively. Cleaved caspase-3↑, caspase-9↑, PARP↑, ΔΨm↓, cyto c↑, Mcl-1↓, p62↑, LC3-II↑, LAMP1↑	Induces autophagy and apoptosis	[60]
Cervical cancer	HeLa	10 μM	Autophagic flux↑, p62↓, LC3-II↑, p70S6K↓, ULK1↑, mTOR↓	Induces autophagy	[57]
		5, 10, 15, 20 μM	LC3-II↑, ROS↑, SOD↑, CAT↑, ΔΨm↓, cyto c↑, caspase-3↑	Induces autophagy, apoptosis, and oxidative stress	[59]
	SiHa, HeLa	2, 4, 6 μM	migration↓, invasion↓, uPA↓, p-p38↓, SOX2↓, OCT4↓, CD49f↓, Nanog↓	Inhibits migration and invasion	[73]
Lung cancer	A549, H1299	20 μM	IC_50_ = 15.33 and 20.95 μM, respectively. Cleaved caspase-3↑, caspase-8↑, caspase-9↑, PARP↑, Cyto c↑, AIF↑, EndoG↑, Bax↑, LC3-II↑, p-AMPK↑, ERK1/2↓	Induces autophagy and apoptosis	[61]
	A549	3, 6, 9 μM	Apoptosis↑, migration↓, invasion↓, MMP-2↓, MMP-9↓, p-ERK1/2↓, p-FAK↓, p-Src↓, β-catenin↓	Induces apoptosis and inhibits migration and invasion	[74]
	A549/Taxol, A2780/Taxol	2, 4, 8 μM	IC_50_ = 5.12 and 4.64 μM, respectively. Apoptosis↑, Bax↑, Bcl-2↓, PARP↓, PI3K↓, AKT↓, p-AKT↓, mTOR↓, Ras↓, Raf↓, MEK↓, ERK↓, p-ERK↓	Induces apoptosis and reverses drug resistance	[90]
	A549, H1299	1, 2, 4 μM	Apoptosis↑, Cell cycle G2/M arrest, migration↓, vimentin↓, Snail-2↓, Snail-1↓, MMP-9↓, E-cadherin↑, ROS↑, MDA↑, iron accumulation↑, HMOX-1↑, FTL↓, GPX4↓, SLC40A1↓, SLC7A11↓	Induces apoptosis, causes cell cycle arrest, inhibits migration, and induces ferroptosis	[103]
Melanoma	A375-S2	2, 4, 6 μM	Cell cycle G1 arrest, cleaved caspase-3↑, LC3-II↑, Beclin 1↑, p-JNK↑, p-ERK↑	Causes cell cycle arrest and induces autophagy and apoptosis	[62]
	B16-F10	10, 50, 100, 200 nM	migration↓, invasion↓, COX-2↓, PGE2↓, EP2↓, EP4↓, p65↓, IKKα↓, IκBα↓	Inhibits migration and invasion	[70]
Bone cancer	143-B, HOS	2, 4, 6 μM	F-actin↓, migration↓, invasion↓, integrin-αv↓, integrin-β3↓, p-FAK, p-Src↓, TESK1↑, p-cofilin↑	Inhibits migration and invasion	[67]
	MG63	6 μM TSAIII + 250 μM Rg1	Apoptotic rate↑, caspase-3↑, migration↓, MMP-2↓, MMP-9↓, p-ERK↓, p-JNK↓, p-p38↓, p-CREB↓, β-catenin↓	Induce apoptosis and inhibits migration	[79]
	MG63	3, 6 μM	Migration↓, invasion↓, MMP-2↓, MMP-9↓, p-FAK↓, p-Src↓, p-ERK↓, p-JNK↓, p-p38↓, p-CREB↓, β-catenin↓, cleaved PARP↑	Inhibits migration and invasion and induces apoptosis	[80]
Renal cancer	786-O, A-498	2, 4, 6 μM	CTSC↓, migration↓, invasion↓, p-PI3K↓, p-AKT↓, miR-129-5p↑	Inhibits migration and invasion	[71]

Note: ↑ indicates up-regulation, and ↓ indicates down-regulation.

## 5. Perspectives and Limitations

TSAIII, as a bioactive steroid saponin from *A. asphodeloides*, has potential anticancer effects in various cancer types. It regulates multiple cellular processes, including cellular proliferation, cell cycle, apoptosis, autophagy, migration, invasion, angiogenesis, multidrug resistance, inflammation, and oxidative stress, by upregulating or downregulating the expression of several key proteins that are involved in several signaling pathways. However, the signaling pathways mediated by TSAIII might be various due to the difference in cancer cell lines, models, and concentrations. It is challenging to investigate the alterations of a specific protein in different experimental models. It should be noted that TSAIII at a high concentration might potentially induce hepatotoxicity due to its activation of oxidative stress. Hopefully, other components in Zhimu, such as norathyriol and mangiferin, could act as antioxidants and exhibit hepatoprotective activity indirectly. TSAIII can enhance the anticancer effects of several chemotherapy agents, such as ginsenoside Rg1, Rb1, and Rc. The synergistic effects of TSAIII are promising, and related clinical trials should be investigated. Due to the hydrophobicity and low bioavailability of TSAIII, effective delivery systems have been developed. Particularly, the tissue/cell-targeted delivery of TSAIII can significantly improve its anticancer effects. More efforts on the pharmacological activities of TSAIII are still needed.

Several patents have been developed. For example, a preparation method for TSAIII has been developed by converting TSBII into TSAIII [106]. TSAIII has been prepared as a medicine to inhibit *Haemophilus parasuis* with a minimum inhibitory concentration (MIC) of 512 μg/mL and a minimum bactericidal concentration of 1024 μg/mL [107]. Another patent reported that TSAIII both prevented and treated nonalcoholic fatty liver disease (NAFLD) by reducing lipid synthesis, enhancing fatty acid oxidation, and upregulating gene expression in relation to mitochondrial functions [108]. TSAIII has been developed as an antihuman rhabdomyosarcoma drug, which can inhibit cell proliferation with a median inhibitory concentration for 24 h at 4.28 μM [109].

There are some limitations to this review article. The pharmacological activities of TSAIII are multiple. However, the main interest of this article is merely in the prevention and treatment of cancers. TSAIII and sarsasapogenin exhibit efficacy in inhibiting tumor progression and can be synthetically modified to improve their pharmacological effects. Some important insights into the derivatives of TSAIII and sarsasapogenin are absent. In addition, experimental data in recently published articles still need further validation.

## 6. Conclusions

*A. asphodeloides* has been used to treat various diseases for centuries, and TSAIII is one of the bioactive compounds responsible for the pharmacological effects of *A. asphodeloides*. TSAIII also has multiple pharmacological activities and could be potentially developed as an anticancer agent. Mechanically, TSAIII inhibits cell proliferation, causes cell cycle arrest, induces apoptosis, mediates autophagy, attenuates migration and invasion, blocks angiogenesis, and improves multidrug resistance in various cancers. However, more efforts are still needed to avoid its shortcomings. TSAIII could be a novel agent for treating cancers in the clinic in the future.

## Data Availability

The data used to support the findings of this study are included within the article.
